# Adherence to a Healthy Lifestyle in Association With Microvascular Complications Among Adults With Type 2 Diabetes

**DOI:** 10.1001/jamanetworkopen.2022.52239

**Published:** 2023-01-26

**Authors:** Gang Liu, Yanping Li, An Pan, Yang Hu, Siyu Chen, Frank Qian, Eric B. Rimm, JoAnn E. Manson, Meir J. Stampfer, Giorgio Giatsidis, Qi Sun

**Affiliations:** 1 Department of Nutrition and Food Hygiene, Hubei Key Laboratory of Food Nutrition and Safety, Ministry of Education Key Laboratory of Environment and Health, State Key Laboratory of Environmental Health (Incubating), School of Public Health, Tongji Medical College, Huazhong University of Science and Technology, Wuhan, China; 2 Department of Nutrition, Harvard T.H. Chan School of Public Health, Boston, Massachusetts; 3 Department of Epidemiology and Biostatistics, School of Public Health, Tongji Medical College, Huazhong University of Science and Technology, Wuhan, China; 4 Department of Endocrinology and Metabolism, Suzhou Dushu Lake Hospital (Dushu Lake Hospital Affiliated to Soochow University), Suzhou, Jiangsu, China; 5 Department of Medicine, Beth Israel Deaconess Medical Center, Harvard Medical School, Boston, Massachusetts; 6 Department of Epidemiology, Harvard T.H. Chan School of Public Health, Boston, Massachusetts; 7 Channing Division of Network Medicine, Department of Medicine, Brigham and Women’s Hospital and Harvard Medical School, Boston, Massachusetts; 8 Division of Preventive Medicine, Department of Medicine, Brigham and Women’s Hospital and Harvard Medical School, Boston, Massachusetts; 9 Department of Surgery, Brigham and Women’s Hospital and Harvard Medical School, Boston, Massachusetts; 10 Joslin Diabetes Center, Boston, Massachusetts

## Abstract

**Importance:**

The association between an overall healthy lifestyle and the subsequent risk of microvascular complications among patients with diabetes remains unclear.

**Objective:**

To examine the association between adherence to a healthy lifestyle before and after diabetes diagnosis and the risk of subsequent microvascular complications among adults with diabetes.

**Design, Setting, and Participants:**

This prospective cohort study included incident patients with type 2 diabetes who were free of cardiovascular disease and cancer at the time of diabetes diagnosis and completed the diabetes supplementary questionnaires in the Nurses’ Health Study (in 2000 and 2005) and the Health Professionals Follow-Up Study (in 2000, 2004, and 2008) in the US. Data were analyzed from April to August 2021.

**Exposures:**

Diet and lifestyle factors before and after diabetes diagnosis were assessed by validated questionnaires. A healthy lifestyle consisted of nonsmoking, having a healthy body weight (a body mass index of ≥18.5 or <25), engaging in moderate-to-vigorous physical activity (≥150 minutes per week), consuming a high-quality diet (top 40th percentile of the Alternative Healthy Eating Index), and moderate alcohol drinking (5-15 g/d for women and 5-30 g/d for men).

**Main Outcomes and Measures:**

Physician-diagnosed microvascular complications including diabetic neuropathy, retinopathy, nephropathy, and foot disorders were self-reported at questionnaire surveys.

**Results:**

A total of 7077 patients with type 2 diabetes were included in the cohort (4982 women in NHS and 2095 men in HPFS, mean [SD] age 61 [8.8], 94.2% White). During follow-up, 2878 patients developed microvascular complications. After multivariable adjustment, adherence to a healthy lifestyle before and after diabetes diagnosis were both associated with a lower risk of developing microvascular complications. The relative risk (RR) for participants with 4 or more low-risk lifestyle factors before diabetes diagnosis compared with zero was 0.73 (95% CI, 0.60-0.91) for any microvascular complications, 0.71 (95% CI, 0.54-0.93) for diabetic neuropathy, 0.76 (95% CI, 0.57-1.01) for diabetic retinopathy, 0.42 (95% CI, 0.23-0.79) for diabetic nephropathy, and 0.60 (95% CI, 0.35-1.00) for diabetic foot disorders. Similar results were observed for adherence to a healthy lifestyle after diabetes diagnosis, with an RR of 0.68 (95% CI, 0.55-0.83) for any microvascular complications, 0.67 (95% CI, 0.51-0.88) for diabetic neuropathy, 0.65 (95% CI, 0.48-0.86) for diabetic retinopathy, 0.57 (95% CI, 0.34-0.98) for diabetic nephropathy, and 0.62 (95% CI, 0.37-1.05) for diabetic foot disorders. In addition, greater improvement in lifestyle factors from before to after diabetes diagnosis was also significantly associated with a lower risk of neuropathy or total microvascular complications. Each increment in number of low-risk lifestyle factors was associated with a 6% (RR, 0.94; 95% CI, 0.90-0.98) lower risk for any microvascular complications and a 9% (RR, 0.91; 95% CI, 0.86-0.96) lower risk for diabetic neuropathy. Consistent results were observed when analyses were stratified by age at diabetes diagnosis, sex/cohort, or lifestyle factors before diabetes diagnosis.

**Conclusions and Relevance:**

In this cohort study, adhering to an overall healthy lifestyle was associated with a significantly lower risk of microvascular complications among individuals with diabetes. These findings suggest substantial reduction in the burden of microvascular complications associated with adopting a healthy lifestyle among patients with type 2 diabetes.

## Key Points

**Question:**

Is adherence to a healthy lifestyle before and after type 2 diabetes (T2D) diagnosis, along with changes in lifestyle from before to after diabetes diagnosis, associated with the risk of microvascular complications among participants with T2D?

**Findings:**

In 2 large prospective cohort studies among 7077 individuals with incident T2D, an overall healthy lifestyle including high-quality diet, nonsmoking, healthy body weight, moderate to vigorous physical activity, and moderate alcohol drinking, both before and after diabetes diagnosis, was associated with a lower risk of microvascular complications. In addition, greater improvements in lifestyle factors from before to after diabetes diagnosis were also associated with a lower risk of microvascular events.

**Meaning:**

These findings suggest a substantial role of lifestyle modification in the prevention of microvascular complications among individuals with T2D and lend support for the American Diabetes Association guidelines that recommend lifestyle improvements for individuals with diabetes.

## Teaser

This cohort study evaluates the association between adherence to a healthy lifestyle and microvascular complications among adults with type 2 diabetes.

## Introduction

Type 2 diabetes (T2D) is a global public health problem with a substantial economic burden on health systems.^1^ Diabetes complications are the leading cause of premature death among individuals with T2D. In addition to macrovascular complications such as cardiovascular disease (CVD), microvascular complications including diabetic nephropathy, retinopathy, and neuropathy have become a substantial burden for patients with diabetes .^2^ For instance, diabetic kidney disease occurs in 20% to 40% of people with diabetes.^3^ The estimated prevalence of diabetic retinopathy was 28.5% among US adults with diabetes in 2005 to 2008.^4^ Thus, it is imperative to identify cost-effective strategies to prevent or delay the development of microvascular complications among patients with diabetes.

In addition to the use of antihyperglycemic agents,^5^ lifestyle modification has played a fundamental role in the management of diabetes. Healthy lifestyle practices, including nonsmoking, maintaining a healthy body weight, engaging in moderate-to-vigorous intensity physical activity, eating a high-quality diet, and avoiding excessive alcohol intake, have been associated with a lower risk of cardiometabolic diseases and mortality in general populations^6,7,8^ or individuals at a high risk of diabetes.^9,10,11,12,13^ However, the evidence regarding the association between combined healthy lifestyle before and after diabetes diagnosis and the subsequent risk of microvascular complications remains scarce. In addition, it remains unclear whether improvements in lifestyle practice from before to after diabetes diagnosis are associated with risks of microvascular events. To fill these knowledge gaps, we prospectively investigated adherence to a healthy lifestyle before and after T2D diagnosis, as well as changes in lifestyle from before to after diabetes diagnosis, in association with the risk of microvascular complications among participants with T2D from 2 large prospective cohort studies.

## Methods

### Study Population

The Nurses’ Health Study (NHS), initiated in 1976, is a prospective cohort study that enrolled 121 700 US female nurses aged 30 to 55 years.^14^ The Health Professionals Follow-Up Study (HPFS), established in 1986, is a prospective cohort study that enrolled 51 529 US male health professionals aged 40 to 75 years.^15^ The NHS and HPFS cohorts were observed using similar methods. In both cohorts, information on diet, lifestyle, medical history, and newly diagnosed diseases was updated every 2 to 4 years through validated questionnaires.^16^ The response rate was approximately 90% in each 2-year cycle for both cohorts. Full details of these 2 prospective cohorts have been published previously.^17,18^

The current study included men and women with incident T2D who were diagnosed during follow-up (1980-2005 for NHS and 1986-2008 for HPFS) and also completed supplementary questionnaires regarding their diabetes treatments and diabetes-related complications (in years 2000 and 2005 in NHS, and 2000, 2004, and 2008 in HPFS). Participants were excluded if they had existing T2D, CVD, or cancer at baseline, had CVD or cancer before T2D diagnosis during follow-up, or had missing information on body mass index (BMI; calculated as weight in kilograms divided by height in meters squared), smoking, alcohol, physical activity, or food frequency questionnaire (FFQ) at the cycle before diabetes diagnosis. To ensure sufficient power to evaluate the association between lifestyle factors and diabetes complications, we pooled the participants from the 2 cohorts in the absence of heterogeneity in results between the 2 cohorts. The current study was approved by the institutional review boards at the Harvard T.H. Chan School of Public Health and Brigham and Women’s Hospital, and the return of the questionnaires was considered implied consent. This study followed the Strengthening the Reporting of Observational Studies in Epidemiology (STROBE) reporting guideline.

### Assessment of Lifestyle Factors

A validated semiquantitative FFQ with approximately 131 food items was administered every 2 to 4 years in both cohorts.^19,20^ Alcohol consumption was obtained from the FFQ, with separate questions for mean daily consumption of spirits, beer, and wine over the past year.^18^ Information on smoking status and body weight was updated via biennial questionnaires. In a small validation study, self-reported body weight was highly correlated with staff-measured body weight (*r* = 0.97).^21^ Physical activity was updated every 2 years through a validated questionnaire,^22^ inquiring about the mean time spent on various activities. Information on potential confounders, including age, self-reported race and ethnicity (race and ethnicity were assessed as basic demographic variables; categories were White and other race and ethnicity), presence of hypertension or hypercholesterolemia, use of antihypertensive or cholesterol-lowering drugs, use of aspirin, and use of multivitamins, was obtained via biennial questionnaires.

### Definition of Low-risk Lifestyle

In the present study, 5 modifiable lifestyle-related factors were included: diet, body weight, smoking status, alcohol consumption, and physical activity. Diet quality was assessed in both cohorts with the use of the 2010 Alternate Healthy Eating Index.^23^ Ten components of the index were included in the diet quality score: high consumption of vegetables, fruits, nuts, whole grains, polyunsaturated fatty acids, and long-chain omega-3 fatty acids and low consumption of red and processed meats, sugar sweetened beverages, trans fat, and sodium. Each component was scored ranging from 0 to 10 according to consumption level, with 10 indicating adherence to the recommended levels of servings per day. A healthy diet was defined as a diet score in the upper 40th percentile of the study population.^16,24^ Healthy body weight was defined as BMI of 18.5 or more and less than 25. For smoking, we defined the low-risk category as noncurrent smoking. Low-risk alcohol consumption was defined as 5 to 15 grams per day for women and 5 to 30 grams per day for men.^25^ Regular physical activity was defined as more than 150 minutes per week of moderate-to-vigorous activities which require the expenditure of 3 metabolic equivalents or more per hour.^25^

Participants received 1 point if they met the criterion for each low-risk lifestyle factor, and 0 points otherwise, with a final low-risk lifestyle score ranging from 0 to 5 (higher score corresponding to a healthier lifestyle).^25^ In a sensitivity analysis, similar results were observed when we assigned weights to each low-risk factor according to the β coefficients from multivariable regression models with diabetes microvascular complications as the outcome.

### Ascertainment of T2D

Participants who reported having a physician’s diagnosis of diabetes were further confirmed by a validated diagnostic questionnaire regarding diagnostic tests, symptoms, and hypoglycemic therapy. We applied the National Diabetes Data Group criteria to define T2D before 1997, and the American Diabetes Association criteria to diagnose T2D after 1997. In our validation studies, 98% of the diabetes cases confirmed by the diagnostic questionnaires were reconfirmed by medical record review in NHS and 97% in HPFS, as previously reported.^26,27^

### Assessment of Diabetes Complications

A supplementary questionnaire regarding diabetes therapy and complications was administered in years 2000 and 2005 in NHS, and 2000, 2004, and 2008 in HPFS. The questionnaire was modified according to the National Health Interview Survey questionnaire, which was designed by Harris et al.^28,29^ Patients with T2D were asked whether they were told by a physician that they had diabetic microvascular complications, including “neuropathy due to diabetes,” “back of eyes/retina affected by diabetes,” “kidney disease related to your diabetes,” “skin ulcer due to diabetes,” or “amputation of toe, foot, leg.” An answer of yes to any of these questions was considered to have diabetic microvascular complications, including diabetic neuropathy, retinopathy, nephropathy, or foot disorders.

### Statistical Analysis

In the current study, we focused on lifestyle factors before (approximately 2-4 years before diagnosis) and after diabetes diagnosis (approximately 2-4 years after diagnosis) associated with subsequent risk of developing diabetic microvascular complications. Due to the high incidence of microvascular complications in the current study population, log-Poisson models were applied to calculate relative risks (RRs) and 95% CIs for the associations of an overall healthy lifestyle with the risk of total microvascular complications, diabetic neuropathy, diabetic retinopathy, diabetic nephropathy, and diabetic foot disorders. Lifestyle factors before diabetes diagnosis were obtained from the most recent questionnaires before diabetes diagnosis. Lifestyle factors after diabetes diagnosis were estimated from the questionnaires right after diabetes diagnosis. Changes in lifestyle score from before to after diabetes diagnosis were defined as postdiabetes low-risk lifestyle scores minus prediabetes low-risk lifestyle scores. Linear trend was tested by treating the lifestyle score as a continuous variable. In the multivariable model, we adjusted for age at diabetes diagnosis (years), sex (men or women), ethnicity (White or non-White), total energy intake (in quartiles), aspirin use (yes or no), presence of hypertension or hypercholesterolemia (yes or no), and use of antihypertensive or cholesterol-lowering drugs (yes or no). For the analysis of changes in lifestyle from before to after diabetes diagnosis, healthy lifestyle score before diabetes diagnosis was further adjusted for in the multivariable model.

Stratified analyses were conducted by age at diabetes diagnosis (<60 years or ≥60 years), sex/cohort (women/NHS or men/HPFS), and the lifestyle score before diabetes diagnosis (<2 or ≥2). The *P* values for the product terms between lifestyle score and stratification variables were used to assess the significance of interactions. Several sensitivity analyses were also conducted to test the robustness of the findings. First, despite the relative homogeneity of socioeconomic status in NHS and HPFS, participants’ perception on their standing in US society (top 20%, 30%, 40%, 50%, or >50%) and educational attainment (registered nurse, bachelor’s degree, master’s degree or higher, or others) were further adjusted (only in the NHS). Second, we also examined the associations of different combinations of low-risk factors with risk of microvascular complications. Third, we further excluded the participants with multiple complications when examining the association of lifestyle factors with individual microvascular complication. Fourth, for the analysis of change in lifestyle factors before and after the diagnosis of diabetes, patients who did not change lifestyles before and after diabetes were used as the reference group.

All statistical analyses were performed with SAS software, version 9.4 (SAS Institute Inc). Two-sided *P* < .05 was considered statistically significant. Data were analyzed from April to August 2021

## Results

After exclusions, 7077 participants (4982 women in NHS and 2095 men in HPFS, mean [SD] age 61 [8.8], 94.2% White) with incident T2D were included in the final analysis. The characteristics of patients with diabetes at diagnosis according to microvascular complications are shown in Table 1. Compared with patients without microvascular complications, patients with microvascular complications tended to be younger and smokers, and were more likely to have higher BMI, lower physical activity, and lower dietary quality. The proportion of participants with 0, 1, 2, 3, or 4 or more low-risk lifestyle factors at diabetes diagnosis was 8.5%, 33.5%, 35.4%, 16.9%, and 5.7%, respectively. eTable 1 in Supplement 1 displays the characteristics of patients with diabetes at diagnosis in men (HPFS) and women (NHS).

**Table 1.** Basic Characteristics of Patients With Diabetes at Diagnosis According to Absence or Presence of Subsequent Microvascular Complications^a^

Characteristic	Patients with microvascular complications, No. (%)	
	Yes (n = 2878)	No (n = 4199)
Age, mean (SD), y	60.0 (8.9)	61.4 (8.7)
Sex		
Male	927 (32.2)	1168 (27.8)
Female	1951 (67.8)	3031 (72.2)
Body mass index, mean (SD)^b^	31.0 (6.0)	30.6 (5.7)
Physical activity, median (IQR), Met-h/wk	7.6 (2.3-20.1)	8.5 (2.7-21.0)
Alcohol consumption, median (IQR), g/d	0.9 (0-4.7)	0.9 (0-4.7)
Alternate Healthy Eating Index, mean (SD)	44.3 (11.3)	45.7 (10.7)
Total energy intake, mean (SD), kcal/d	1839.9 (613.9)	1824.6 (588.3)
White	2719 (94.5)	3947 (94.0)
Smoking status		
Never	1189 (41.3)	1855 (44.2)
Past	1322 (45.9)	1910 (45.5)
Current	367 (12.8)	434 (10.3)
Hypertension	1788 (62.1)	2538 (60.4)
Hypercholesterolemia	1331 (46.3)	2241 (53.4)
Family history of myocardial infarction	936 (32.5)	1347 (32.1)
Aspirin use	1407 (48.9)	1965 (46.8)
Use of antihypertensive drugs	1204 (41.8)	1954 (46.5)
Use of cholesterol-lowering drugs	368 (12.8)	733 (17.5)

Abbreviation: Met-h, metabolic hours.

^a^ Diabetic microvascular complications include diabetic neuropathy, retinopathy, nephropathy, or foot disorders. The information was obtained from the questionnaire in which the participants first reported incident diabetes.

^b^ Body mass index is calculated as weight in kilograms divided by height in meters squared.

A total of 2878 incident cases of diabetic microvascular complications were documented during follow-up, including 1796 cases of diabetic neuropathy, 1415 cases of diabetic retinopathy, 383 cases of diabetic nephropathy, and 452 cases of diabetic foot disorders. Adherence to a healthy lifestyle before diabetes diagnosis was significantly associated with a lower risk of developing microvascular complications (Table 2). After multivariable adjustment, comparing participants with 0 low-risk lifestyle factors, the participants with 4 or more low-risk lifestyle factors had an RR of 0.73 (95% CI, 0.60- 0.91) for any microvascular complications (*P* for trend = .006), 0.71 (95% CI, 0.54-0.93) for diabetic neuropathy (*P* for trend = .01), 0.76 (95% CI, 0.57-1.01) for diabetic retinopathy (*P* for trend = .09), 0.42 (95% CI, 0.23-0.79) for diabetic nephropathy (*P* for trend = .01), and 0.60 (95% CI, 0.35-1.00) for diabetic foot disorders (*P* for trend = .049).

**Table 2.** Risk of Diabetic Microvascular Complications According to Number of Low-risk Lifestyle Factors Before Diabetes Diagnosis

Complication	Relative Risk by No. of low-risk lifestyle factors (95% CI)^a^					*P* value for trend
	0 (n = 601)	1 (n = 2371)	2 (n = 2505)	3 (n = 1193)	≥4 (n = 407)	
Any diabetic microvascular complications						
Cases, No.	301	971	1014	453	139	NA
Age-adjusted model	1 [Reference]	0.85 (0.75-0.97)	0.86 (0.75-0.98)	0.82 (0.70-0.95)	0.73 (0.59-0.90)	.007
Multivariable-adjusted model	1 [Reference]	0.89 (0.78-1.02)	0.89 (0.78-1.01)	0.84 (0.72-0.97)	0.73 (0.60-0.91)	.006
Diabetic neuropathy						
Cases, No.	183	621	636	277	79	NA
Age-adjusted model	1 [Reference]	0.90 (0.76-1.06)	0.89 (0.75-1.05)	0.83 (0.69-1.01)	0.70 (0.53-0.91)	.009
Multivariable-adjusted model	1 [Reference]	0.92 (0.78-1.09)	0.91 (0.77-1.07)	0.85 (0.70-1.03)	0.71 (0.54-0.93)	.01
Diabetic retinopathy						
Cases, No.	162	464	498	220	71	NA
Age-adjusted model	1 [Reference]	0.78 (0.65-0.93)	0.82 (0.68-0.98)	0.78 (0.64-0.97)	0.74 (0.56-0.99)	.12
Multivariable-adjusted model	1 [Reference]	0.87 (0.72-1.04)	0.89 (0.74-1.07)	0.83 (0.68-1.03)	0.76 (0.57-1.01)	.09
Diabetic nephropathy						
Cases, No.	49	130	128	63	13	NA
Age-adjusted model	1 [Reference]	0.72 (0.51-1.00)	0.69 (0.49-0.96)	0.73 (0.50-1.08)	0.44 (0.24-0.82)	.04
Multivariable-adjusted model	1 [Reference]	0.79 (0.56-1.11)	0.73 (0.52-1.03)	0.75 (0.51-1.10)	0.42 (0.23-0.79)	.01
Diabetic foot disorders						
Cases, No.	56	150	159	67	20	NA
Age-adjusted model	1 [Reference]	0.71 (0.52-0.97)	0.72 (0.53-0.99)	0.66 (0.46-0.94)	0.57 (0.34-0.96)	.043
Multivariable-adjusted model	1 [Reference]	0.78 (0.57-1.07)	0.78 (0.57-1.07)	0.70 (0.48-1.01)	0.60 (0.35-1.00)	.049

Abbreviation: NA, not applicable.

^a^ Low-risk lifestyle factors: nonsmoking, healthy body weight (body mass index [calculated as weight in kilometers divided by height in meters squared] at diagnosis, 18.5 to <25), moderate to vigorous physical activity (>150 minutes per week), high-quality diet (top two-fifths of Alternative Healthy Eating Index), and moderate alcohol consumption (5-15 grams per day for female and 5-30 grams per day for male).

Multivariable model was adjusted for age at diagnosis (years), sex (men or women), race (White or other race and ethnicity), total energy intake (in quartiles), aspirin use (yes or no), presence of hypertension (yes or no), presence of hypercholesterolemia (yes or no), use of antihypertensive drugs (yes or no), and use of cholesterol-lowering drugs (yes or no).

Similar results were observed for adherence to a healthy lifestyle after diabetes diagnosis (Table 3). Comparing participants with 0 low-risk lifestyle factors, participants with 4 or more low-risk lifestyle factors had an RR of 0.68 (95% CI, 0.55-0.83) for any microvascular complications (*P* for trend<.001), 0.67 (95% CI, 0.51-0.88) for diabetic neuropathy (*P* for trend <. 001), 0.65 (95% CI, 0.48-0.86) for diabetic retinopathy (*P* for trend = .09), 0.57 (95% CI, 0.34-0.98) for diabetic nephropathy (*P* for trend = .20), and 0.62 (95% CI, 0.37-1.05) for diabetic foot disorders (*P* for trend = .03).

**Table 3.** Risk of Diabetic Microvascular Complications According to Number of Low-risk Lifestyle Factors After Diabetes Diagnosis

Complication	Relative risk by No. of low-risk lifestyle factors (95% CI)^a^					*P* value for trend
	0 (n = 497)	1 (n = 2496)	2 (n = 2391)	3 (n = 1236)	≥4 (n = 457)	
Any diabetic microvascular complications						
Cases, No.	255	1040	964	473	146	NA
Age-adjusted model	1 [Reference]	0.85 (0.74-0.97)	0.84 (0.73-0.97)	0.80 (0.68-0.93)	0.67 (0.54-0.82)	<.001
Multivariable-adjusted model	1 [Reference]	0.90 (0.78-1.04)	0.88 (0.76-1.02)	0.82 (0.70-0.97)	0.68 (0.55-0.83)	<.001
Diabetic neuropathy						
Cases, No.	153	685	610	263	85	NA
Age-adjusted model	1 [Reference]	0.94 (0.79-1.13)	0.90 (0.75-1.07)	0.75 (0.61-0.92)	0.66 (0.51-0.87)	<.001
Multivariable-adjusted model	1 [Reference]	0.97 (0.81-1.16)	0.92 (0.77-1.11)	0.77 (0.63-0.95)	0.67 (0.51-0.88)	<.001
Diabetic retinopathy						
Cases, No.	146	481	455	260	73	NA
Age-adjusted model	1 [Reference]	0.72 (0.60-0.87)	0.73 (0.61-0.89)	0.82 (0.67-1.01)	0.63 (0.47-0.84)	.18
Multivariable-adjusted model	1 [Reference]	0.83 (0.68-1.00)	0.82 (0.67-0.99)	0.89 (0.72-1.09)	0.65 (0.48-0.86)	.09
Diabetic nephropathy						
Cases, No.	45	124	125	68	21	NA
Age-adjusted model	1 [Reference]	0.60 (0.42-0.85)	0.65 (0.46-0.92)	0.69 (0.47-1.01)	0.58 (0.34-0.98)	.35
Multivariable-adjusted model	1 [Reference]	0.69 (0.48-0.98)	0.71 (0.50-1.01)	0.74 (0.50-1.09)	0.57 (0.34-0.98)	.20
Diabetic foot disorders						
Cases, No.	46	164	152	67	23	NA
Age-adjusted model	1 [Reference]	0.75 (0.54-1.05)	0.74 (0.53-1.04)	0.64 (0.43-0.94)	0.60 (0.36-0.99)	.03
Multivariable-adjusted model	1 [Reference]	0.86 (0.61-1.20)	0.82 (0.58-1.16)	0.70 (0.47-1.03)	0.62 (0.37-1.05)	.03

Abbreviation: NA, not applicable.

^a^ Low-risk lifestyle factors: nonsmoking, healthy body weight (body mass index ≥18.5 to <25 shortly after diagnosis, calculated as weight in kilograms divided by height in meters squared), moderate to vigorous physical activity (>150 minutes per week), high-quality diet (top two-fifths of Alternative Healthy Eating Index), and moderate alcohol consumption (5-15 g/d for female and 5-30 g/d for male).

Multivariable model was adjusted for age at diagnosis (years), sex (men or women), race (White or other race and ethnicity), total energy intake (in quartiles), current aspirin use (yes or no), presence of hypertension (yes or no), presence of hypercholesterolemia (yes or no), use of antihypertensive drugs (yes or no), and use of cholesterol-lowering drugs (yes or no).

In addition, greater improvements in lifestyle factors from before to after diabetes diagnosis were also significantly associated with a lower risk of neuropathy or total microvascular complications (Table 4). Each increment in number of low-risk lifestyle factors was associated with a 6% lower risk for any microvascular complications (RR, 0.94; 95% CI, 0.90-0.98; P = .01) and a 9% lower risk for diabetic neuropathy (RR, 0.91; 95% CI, 0.86-0.96; P = .001).

**Table 4.** Risk of Diabetic Microvascular Complications According to Changes in Lifestyle Factors Before and After Diabetes Diagnosis^a^

Complication	Relative risk per 1 No. increment in low-risk lifestyle (95% CI) (n = 7707)	*P *value
Any diabetic microvascular complications		
Cases, No.	2878	NA
Age-adjusted model	0.98 (0.94-1.02)	.41
Multivariable-adjusted model	0.94 (0.90-0.98)	.01
Diabetic neuropathy		
Cases, No.	1796	NA
Age-adjusted model	0.95 (0.91-1.00)	.07
Multivariable-adjusted model	0.91 (0.86-0.96)	.001
Diabetic retinopathy		
Cases, No.	1415	NA
Age-adjusted model	1.00 (0.95-1.06)	.93
Multivariable-adjusted model	0.97 (0.91-1.03)	.31
Diabetic nephropathy		
Cases, No.	383	NA
Age-adjusted model	1.07 (0.96-1.20)	.20
Multivariable-adjusted model	1.01 (0.90-1.15)	.79
Diabetic foot disorders		
Cases, No.	452	NA
Age-adjusted model	0.99 (0.89-1.09)	.81
Multivariable-adjusted model	0.93 (0.83-1.04)	.20

Abbreviation: NA, not applicable.

^a^ Multivariable model was adjusted for age at diagnosis (years), sex (men or women), race (White or other race and ethnicity), total energy intake (in quartiles), aspirin use (yes or no), presence of hypertension (yes or no), presence of hypercholesterolemia (yes or no), use of antihypertensive drugs (yes or no), use of cholesterol-lowering drugs (yes or no), and number of low-risk lifestyle factors before diabetes diagnosis.

Consistent results were observed when analyses were stratified by age at diabetes diagnosis, sex/cohort, or lifestyle factors before diabetes diagnosis, although with reduced power some of the results did not reach statistical significance (eTables 2 and 3 in Supplement 1). No significant interactions were detected between the strata and low-risk lifestyle score (all *P* for interaction >.10). Similar findings were demonstrated when further adjusting for participants’ perception on their standing in US society and educational attainment (in the NHS), when different combinations of lifestyle factors were examined in relation to risk of microvascular complications (eTables 4 and 5 in Supplement 1), when further excluding the participants with multiple complications, or when using patients who did not change lifestyles before and after diabetes as the reference group.

## Discussion

In 2 large prospective cohort studies among individuals with incident T2D, we found that an overall healthy lifestyle, including high-quality diet, nonsmoking, healthy body weight, moderate to vigorous physical activity, and moderate alcohol drinking, both before and after diabetes diagnosis, was associated with a lower risk of microvascular complications. In addition, greater improvements in lifestyle factors from before to after diabetes diagnosis were also associated with a lower risk of microvascular events.

Although abundant evidence has indicated that a healthy lifestyle is associated with a lower risk of cardiometabolic diseases in general populations,^6,7,8^ several lifestyle intervention trials among individuals at a high risk of diabetes or CVD demonstrated mixed findings regarding effects of lifestyle modification on microvascular complications.^30,31^ For instance, in the China Da Qing Diabetes Prevention Study among 577 participants with impaired glucose tolerance, Gregg et al^30^ found that lifestyle interventions through diet and exercise for 6 years resulted in a 47% reduction in the incidence of severe retinopathy, but not for nephropathy or neuropathy. In the Diabetes Prevention Program Outcomes Study^31^ among individuals at a high risk of diabetes, intensive lifestyle intervention (including diet and physical activity) or metformin did not lower the prevalence of microvascular events compared with placebo after a 15-year follow-up in the whole study population, although a 21% reduction with lifestyle intervention compared with placebo was observed in women. Of note, microvascular complications were not predefined primary outcomes in these trials.

Prior observational studies largely focused on individual lifestyle factors (eg, diet, smoking, and physical activity) after diabetes diagnosis and risks of certain microvascular events among individuals with diabetes.^32,33,34,35,36^ For example, Dunkler et al^32^ found that high diet quality (indicated as modified Alternate Healthy Eating index) was associated with a lower incidence or progression of chronic kidney disease after 5.5 years of follow-up among 3088 European participants with diabetes. In a secondary analysis of ADVANCE (Action in Diabetes and Vascular Disease: Preterax and Diamicron Controlled Evaluation) study consisting of 11 140 participants with diabetes, moderate to vigorous physical activity and moderate alcohol consumption were each associated with a 15% lower incidence of microvascular complications after approximately 5 years of follow-up.^34,35^ Evidence regarding the impact of combined healthy lifestyle before and after diabetes diagnosis on the subsequent risk of microvascular complications is limited. In a few multicomponent lifestyle interventions in patients with diabetes, findings were mixed.^37,38,39^ In a post hoc analysis of the PREDIMED (Prevención con Dieta Mediterránea) trial, Mediterranean diet supplemented with extra virgin olive oil resulted in a 43% lower incidence of diabetic retinopathy, but not for diabetic nephropathy, after a median 6 years of follow-up.^37^ In the Look AHEAD (Action for Health in Diabetes) trial enrolling 5145 people with diabetes and overweight or obesity, intensive lifestyle intervention (ie, reducing energy intake and increasing physical activity) significantly reduced the incidence of chronic kidney disease after 8 years of follow-up,^38^ but with no significant effect on physical examination measures of diabetic peripheral neuropathy.^39^ The heterogeneity of these findings may be partially explained by small numbers of cases of microvascular events (eg, 74 diabetic retinopathy and 168 diabetic nephropathy in the PREDIMED trial) and use of unadjudicated events of microvascular complications (eg, Michigan Neuropathy Screening Instrument questionnaire was used to evaluate neuropathy in the Look AHEAD trial).

Evidence regarding whether changes in lifestyle from before to after diabetes diagnosis would yield benefits for lowering subsequent microvascular events is absent. Using 2 large prospective cohorts, we showed for the first time, to our knowledge, that adherence to healthy lifestyle practices both before and after diabetes diagnosis were significantly associated with a lower risk of microvascular complications. Moreover, we further found that improvements in lifestyle factors from before to after diabetes diagnosis were also associated with a lower risk of microvascular events. Overall, our findings together with existing evidence suggest that adopting a healthy diet and lifestyle could aid in the prevention of microvascular complications among people with T2D.

Although the exact mechanisms underlying the observed associations between healthy lifestyle and microvascular complications remains unclear, some studies suggest that diabetes and hyperglycemia might generate a proinflammatory microenvironment that leads to microvascular complications such as diabetic retinopathy, nephropathy, and neuropathy.^40^ In addition, dyslipidemia might induce or exacerbate diabetic nephropathy and retinopathy through alterations in membrane permeability, changes in the coagulation-fibrinolytic system, or destruction of endothelial cells.^41^ Evidence from randomized clinical trials has revealed that adopting a healthy diet or increasing physical activity could substantially reduce inflammatory markers (such as C-reactive protein, interleukin-6, and tumor necrosis factor receptors) and improve blood pressure, lipid profiles, endothelial dysfunction, and insulin resistance.^42,43,44^ Nevertheless, more studies are warranted to clarify the underlying mechanisms.

### Strengths and Limitations

To our knowledge, this is the first study that not only examines the combined dietary and lifestyle factors before and after diabetes diagnosis, but also investigates the changes in lifestyle from before to after diabetes diagnosis, with subsequent risk of microvascular complications, including diabetic neuropathy, retinopathy, nephropathy, and foot disorders among US adults with incident T2D. The strengths of our study also include the fairly large sample size and detailed assessments of dietary and lifestyle factors before and after diabetes diagnosis. In addition, the reliability and validity of self-reported lifestyle and diet among these health professionals are also a strength.^45^

Several limitations should be also acknowledged. First, the study participants were all health professionals and most were non-Hispanic White participants, which may limit the generalizability of these findings to other populations or racial and ethnic groups, although the relative homogeneity may help to minimize confounding by socioeconomic status. Second, lifestyle factors after diabetes were assessed only shortly after the diagnosis of diabetes, and the identification of microvascular complications was according to self-reported physician diagnosis, and the validity of these was not examined. We could not exclude the possibility that health consciousness may lead to an adherence to a healthy lifestyle and a higher chance of reporting a complication, although such a bias would primarily result in attenuation of the risk reductions observed. Additionally, residual confounding or potential confounding by genetic susceptibility, psychosocial stress, or pharmacologic treatment could not be excluded in observational studies.^46^

## Conclusions

In 2 large prospective cohort studies of US adults with incident T2D, adhering to an overall healthy lifestyle before and after diabetes diagnosis were both associated with a substantially lower risk of microvascular complications. In addition, greater improvements in lifestyle factors from before to after diabetes diagnosis were also associated with a lower risk of microvascular events. These findings suggest a substantial role of lifestyle modification in the prevention of microvascular complications among individuals with T2D and lend support for the American Diabetes Association guidelines that recommend lifestyle improvements for individuals with diabetes.

## Notes

Supplement 1. **eTable 1. **Basic Characteristics of Diabetes Patients at Diagnosis in NHS and HPFS **eTable 2. **Stratified Analysis of Diabetic Microvascular Complications According to Number of Low-Risk Lifestyle Factors Before Diabetes Diagnosis **eTable 3. **Stratified Analysis of Diabetic Microvascular Complications According to Number of Low-Risk Lifestyle Factors After Diabetes Diagnosis **eTable 4. **Relative Risk (95% CI) of Diabetic Microvascular Complications According to Different Combinations of Low-Risk Lifestyle Factors Before Diabetes Diagnosis **eTable 5. **Relative Risk (95% CI) of Diabetic Microvascular Complications According to Different Combinations of Low-Risk Lifestyle Factors After Diabetes Diagnosis

Supplement 2.
Data Sharing Statement
