# Supplementary material for: Adherence to a Healthy Lifestyle in Association With Microvascular Complications Among Adults With Type 2 Diabetes
Source: JAMA Netw Open. 2023 Jan 26;6(1):e2252239. doi: 10.1001/jamanetworkopen.2022.52239 (PMC9880795; doi:10.1001/jamanetworkopen.2022.52239)
Supplement: Supplement 1. — eTable 1. Basic Characteristics of Diabetes Patients at Diagnosis in NHS and HPFS eTable 2. Stratified Analysis of Diabetic Microvascular Complications According to Number of Low-Risk Lifestyle Factors Before Diabetes Diagnosis eTable 3. Stratified Analysis of Diabetic Microvascular Complications According to Number of Low-Risk Lifestyle Factors After Diabetes Diagnosis eTable 4. Relative Risk (95% CI) of Diabetic Microvascular Complications According to Different Combinations of Low-Risk Lifestyle Factors Before Diabetes Diagnosis eTable 5. Relative Risk (95% CI) of Diabetic Microvascular Complications According to Different Combinations of Low-Risk Lifestyle Factors After Diabetes Diagnosis [file jamanetwopen-e2252239-s001.pdf]

Supplemental Online Content

Liu G, Li Y, Pan A, et al. Adherence to a healthy lifestyle in association with microvascular complications among adults with type 2 diabetes. *JAMA Netw Open*. 2023;6(1):e2252239. doi:10.1001/jamanetworkopen.2022.52239

eTable 1. Basic Characteristics of Diabetes Patients at Diagnosis in NHS and HPFS

eTable 2. Stratified Analysis of Diabetic Microvascular Complications According to Number of Low-Risk Lifestyle Factors Before Diabetes Diagnosis

eTable 3. Stratified Analysis of Diabetic Microvascular Complications According to Number of Low-Risk Lifestyle Factors After Diabetes Diagnosis

eTable 4. Relative Risk (95% CI) of Diabetic Microvascular Complications According to Different Combinations of Low-Risk Lifestyle Factors Before Diabetes Diagnosis

eTable 5. Relative Risk (95% CI) of Diabetic Microvascular Complications According to Different Combinations of Low-Risk Lifestyle Factors After Diabetes Diagnosis

This supplemental material has been provided by the authors to give readers additional information about their work.

© 2023 Liu G et al. *JAMA Network Open.*

eTable 1. Basic Characteristics of Diabetes Patients at Diagnosis in NHS and HPFS [a]

	**NHS** (n=4,982)	**HPFS** (n=2,095)
Age, years	60.4 (8.9)	61.9 (8.5)
Body Mass Index, kg/m^2	31.5 (6.1)	29.0 (4.5)
Physical activity, Met-hrs/week	6.3 (2.0, 15.9)	15.1 (4.9, 33.8)
Alcohol consumption, g/day	0 (0, 2.1)	3.9 (0, 12.9)
Alternate Healthy Eating Index	44.4 (11.1)	46.7 (10.6)
Total energy intake, kcal/day	1755.1 (562.7)	2010.8 (642.5)
White, %	95.8	90.5
Smoking status, %		
Never	45.1	38.0
Past	42.0	54.4
Current	11.9	7.6
Hypertension, %	65.0	52.0
Hypercholesterolemia, %	51.9	47.0
Family history of MI, %	30.8	35.7
Aspirin use, %	48.3	46.1
Use of antihypertensive drugs, %	46.4	40.3
Use of cholesterol-lowering drugs, %	15.9	14.8

[a] Values are means (SD), median (interquartile range), or %; NHS: Nurses' Health Study (in 2000 and 2005); HPFS: Health Professionals' Follow-Up Study (in 2000, 2004, and 2008); MI: myocardial infarction.

© 2023 Liu G et al. *JAMA Network Open.*

eTable 2. Stratified Analysis of Diabetic Microvascular Complications According to Number of Low-Risk Lifestyle Factors Before Diabetes Diagnosis

	**Number of Low-Risk Lifestyle Factors** [a]					***P* trend**
	**0**	**1**	**2**	**3**	**≥4**	
**Age at diagnosis (years)**						
<60	1.00 (ref)	0.88 (0.75-1.04)	0.85 (0.72-1.00)	0.80 (0.65-0.99)	0.64 (0.46-0.90)	0.004
≥60	1.00 (ref)	0.94 (0.74-1.18)	0.96 (0.76-1.21)	0.89 (0.69-1.13)	0.82 (0.61-1.10)	0.17
**Sex/Cohort**						
Women/NHS	1.00 (ref)	0.91 (0.78-1.07)	0.90 (0.77-1.06)	0.87 (0.72-1.05)	0.75 (0.55-1.03)	0.08
Men/HPFS	1.00 (ref)	0.81 (0.63-1.02)	0.81 (0.65-1.03)	0.74 (0.58-0.95)	0.66 (0.50-0.89)	0.008

Multivariable model was adjusted for age at diagnosis (years), sex (men, women), race (white, non-white), total energy intake (in quartiles), current aspirin use (yes, no), presence of hypertension (yes, no), presence of hypercholesterolemia (yes, no), use of antihypertensive drugs (yes, no), and use of cholesterol-lowering drugs (yes, no). Strata variable was not included in the model when stratifying by itself.

[a] Low-risk lifestyle factors: non-smoking, healthy body weight (18.5≤BMI shortly after diagnosis<25 kg/m^2), moderate to vigorous physical activity (>150 min/week), high quality diet (top two fifths of Alternative Healthy Eating Index), and moderate alcohol consumption (5-15 g/day for female and 5-30 g/day for male).

© 2023 Liu G et al. *JAMA Network Open.*

eTable 3. Stratified Analysis of Diabetic Microvascular Complications According to Number of Low-Risk Lifestyle Factors After Diabetes Diagnosis

	**Number of Low-Risk Lifestyle Factors** [a]					***P* trend**
	**0**	**1**	**2**	**3**	**≥4**	
**Age at diagnosis (years)**						
<60	1.00 (ref)	0.87 (0.74-1.03)	0.90 (0.76-1.06)	0.81 (0.67-0.99)	0.64 (0.47-0.87)	0.01
≥60	1.00 (ref)	1.04 (0.78-1.38)	0.95 (0.72-1.26)	0.91 (0.68-1.22)	0.76 (0.54-1.06)	0.01
**Sex/Cohort**						
Women/NHS	1.00 (ref)	0.95 (0.80-1.12)	0.91 (0.77-1.09)	0.86 (0.70-1.05)	0.66 (0.48-0.92)	0.01
Men/HPFS	1.00 (ref)	0.79 (0.61-1.01)	0.78 (0.61-1.00)	0.74 (0.57-0.96)	0.61 (0.46-0.82)	0.003
**Lifestyle score before diabetes diagnosis**						
<2	1.00 (ref)	0.94 (0.80-1.10)	0.91 (0.77-1.09)	0.84 (0.65-1.10)	0.71 (0.33-1.50)	0.14
≥2	1.00 (ref)	0.80 (0.47-1.36)	0.77 (0.46-1.31)	0.73 (0.43-1.25)	0.60 (0.34-1.04)	0.004

Multivariable model was adjusted for age at diagnosis (years), sex (men, women), race (white, non-white), total energy intake (in quartiles), current aspirin use (yes, no), presence of hypertension (yes, no), presence of hypercholesterolemia (yes, no), use of antihypertensive drugs (yes, no), use of cholesterol-lowering drugs (yes, no), and lifestyle score before diabetes diagnosis. Strata variable was not included in the model when stratifying by itself.

[a] Low-risk lifestyle factors: non-smoking, healthy body weight (18.5≤BMI shortly after diagnosis<25 kg/m^2), moderate to vigorous physical activity (>150 min/week), high quality diet (top two fifths of Alternative Healthy Eating Index), and moderate alcohol consumption (5-15 g/day for female and 5-30 g/day for male).

© 2023 Liu G et al. *JAMA Network Open.*

eTable 4. Relative Risk (95% CI) of Diabetic Microvascular Complications According to Different Combinations of Low-Risk Lifestyle Factors Before Diabetes Diagnosis

	Number of Low-Risk Lifestyle Factors*					*P* trend
	**0**	**1**	**2**	**3**	**≥4**	
**Three low-risk factors (healthy diet, regular physical activity, non-smoking)**						
N	773	2,952	3,352			
Multivariable-adjusted model	1.00 (ref)	0.88 (0.78-0.98)	0.86 (0.76-0.96)	-	-	0.03
**Above three low-risk factors + healthy body weight**						
N	679	2,700	2,549	1,149		
Multivariable-adjusted model	1.00 (ref)	0.89 (0.78-1.00)	0.89 (0.79-1.01)	0.82 (0.70-0.95)	-	0.03
**Above three low-risk factors + moderate alcohol drinking**						
N	676	2,589	2,573	1,239		
Multivariable-adjusted model	1.00 (ref)	0.88 (0.78-1.00)	0.86 (0.76-0.97)	0.80 (0.69-0.92)	-	0.004
**Above three low-risk factors + healthy body weight + moderate alcohol drinking**						
N	301	971	1,014	453	139	
Multivariable-adjusted model	1.00 (ref)	0.89 (0.78-1.02)	0.89 (0.78-1.01)	0.84 (0.72-0.97)	0.73 (0.60-0.91)	0.006

Multivariable model was adjusted for age at diagnosis (years), sex (men, women), race (white, non-white), total energy intake (in quartiles), current aspirin use (yes, no), presence of hypertension (yes, no), presence of hypercholesterolemia (yes, no), use of antihypertensive drugs (yes, no), and use of cholesterol-lowering drugs (yes, no).

© 2023 Liu G et al. *JAMA Network Open.*

eTable 5. Relative Risk (95% CI) of Diabetic Microvascular Complications According to Different Combinations of Low-Risk Lifestyle Factors After Diabetes Diagnosis

	Number of Low-Risk Lifestyle Factors*					*P* trend
	0	1	2	3	⩾4	
**Three low-risk factors (healthy diet, regular physical activity, non-smoking)**						
N	617	3,184	3,276			
Multivariable-adjusted model	1.00 (ref)	0.89 (0.78-1.01)	0.85 (0.74-0.96)	-	-	0.01
**Above three low-risk factors + healthy body weight**						
N	535	2,820	2,417	1,305		
Multivariable-adjusted model	1.00 (ref)	0.89 (0.78-1.02)	0.91 (0.79-1.04)	0.77 (0.66-0.90)	-	0.004
**Above three low-risk factors + moderate alcohol drinking**						
N	566	2,803	2,502	1,206		
Multivariable-adjusted model	1.00 (ref)	0.91 (0.80-1.04)	0.86 (0.75-0.98)	0.77 (0.67-0.90)	-	<0.001
**Above three low-risk factors + healthy body weight + moderate alcohol drinking**						
N	255	1,040	964	473	146	
Multivariable-adjusted model	1.00 (ref)	0.90 (0.78-1.04)	0.88 (0.76-1.02)	0.82 (0.70-0.97)	0.68 (0.55-0.83)	<0.001

Multivariable model was adjusted for age at diagnosis (years), sex (men, women), race (white, non-white), total energy intake (in quartiles), current aspirin use (yes, no), presence of hypertension (yes, no), presence of hypercholesterolemia (yes, no), use of antihypertensive drugs (yes, no), use of cholesterol-lowering drugs (yes, no), and lifestyle score before diabetes diagnosis.

© 2023 Liu G et al. *JAMA Network Open.*
