# Supplementary material for: Adherence to a Healthy Lifestyle in Association With Microvascular Complications Among Adults With Type 2 Diabetes
Source: JAMA Netw Open. 2023 Jan 26;6(1):e2252239. doi: 10.1001/jamanetworkopen.2022.52239 (PMC9880795; doi:10.1001/jamanetworkopen.2022.52239)
Supplement: Supplement 2. — Data Sharing Statement [file jamanetwopen-e2252239-s002.pdf]

Data Sharing Statement

Liu. Adherence to a Healthy Lifestyle in Association With Microvascular Complications Among Adults With Type 2 Diabetes. *JAMA Netw Open*. Published January 26, 2023. doi:10.1001/jamanetworkopen.2022.52239

Data
Data available: No
